# Current status and challenges of planetary health education: a scoping review

**DOI:** 10.1265/ehpm.25-00437

**Published:** 2026-04-23

**Authors:** Yayoi Shoji, Kana Suzuki, Masushi Kohta, Nao Tamai

**Affiliations:** 1 Public Health Unit, Global Cooperation Institute for Sustainable Cities, Yokohama City University, Yokohama, Japan; 2 Graduate School of Health Care and Nursing, Juntendo University, Urayasu, Japan; 3 Department of Nursing, Graduate School of Medicine, Yokohama City University, Yokohama, Japan; 4 Institute of Tropical Medicine, Nagasaki University, Nagasaki, Japan; 5 Research Center for Implementation Nursing Science Initiative, Fujita Health University, Aichi, Japan

**Keywords:** Planetary health, Climate change, Educational programs, Curriculum integration, Scoping review

## Abstract

**Background:**

Climate change poses significant threats to human health, necessitating the integration of environmental protection into public health efforts. Planetary health education (PHE) represents an emerging paradigm connecting human well-being with natural systems; however, its global implementation remains inadequately documented, particularly in environmentally vulnerable regions. This scoping review aimed to investigate how PHE is implemented across educational settings and to identify current challenges and best practices to inform future educational program development for university students, educators, and healthcare professionals.

**Methods:**

Following Arksey and O’Malley’s framework and the PRISMA-ScR guidelines, we systematically searched the following databases for English and Japanese literature published up to September 30, 2025: Web of Science, Cochrane Central Register of Controlled Trials (CENTRAL), CINAHL, Scopus, EMBASE, PubMed, OpenGrey, and Ichushi-Web. Using the Patient–Concept–Context framework, we extracted data on educational programs, implementation methods, challenges, outcomes, and curriculum integration.

**Results:**

Forty-one studies met the inclusion criteria. These studies were predominantly from high-income countries with varying Climate Change Performance Index rankings. Implementation was concentrated in higher education institutions, particularly medical and nursing schools. Educational approaches included lecture-based teaching, group work, practical training, online learning, and simulations. Key content areas were most frequently climate change and health/climate-sensitive care and sustainability/SDG-related topics, whereas pollution/waste and environmental toxins, food systems, and equity/justice-related content were reported less frequently. Primary challenges included difficulties with curriculum integration, time constraints, and insufficient awareness among educators. Reported outcomes demonstrated improvements in knowledge, critical thinking, and motivation for sustainable practices; however, longitudinal impact studies were notably absent.

**Conclusions:**

PHE requires expansion beyond medicine into diverse disciplines and geographical contexts, particularly in low-income countries. Longitudinal studies and randomized controlled trials are needed to assess the long-term educational impact. Successful implementation depends on interdisciplinary approaches, strategic curriculum integration, and educator development programs. These efforts will help address global health challenges through education that connects human health with environmental sustainability.

**Supplementary information:**

The online version contains supplementary material available at https://doi.org/10.1265/ehpm.25-00437.

## Background

Climate change poses a significant threat to human health and well-being, prompting the Centers for Disease Control and Prevention (CDC) in the United States to identify air pollution, extreme weather events, heat waves, water quality deterioration, and increased allergens as potential consequences [1]. Although economic development and human longevity have advanced, modern society has substantially burdened the environment. The concept of planetary health, introduced in 2015 by the Rockefeller Foundation and The Lancet, emphasizes the interconnectedness of human and planetary health, advocating for a holistic approach to improving and sustaining the health of all ecosystems [2, 3].

Planetary health emphasizes the necessity of integrating environmental protection into public health efforts for a sustainable future. For example, the Rockefeller Foundation–Lancet Commission on planetary health warned that human-driven environmental changes (e.g., climate change and biodiversity loss) can undermine the foundations of health and reverse hard-won public health gains [3]. Several indicators are used to evaluate climate change and environmental burdens. The United Nations Development Program (UNDP) introduced the Planetary Pressures–adjusted Human Development Index (PHDI) in its 2020 Human Development Report, which extends the Human Development Index (HDI) by considering environmental impacts [4]. Europe leads the PHDI rankings, with Japan ranked 10th globally, whereas Southeast Asian countries such as Thailand and Malaysia lag, suggesting insufficient planetary health initiatives. The Climate Change Performance Index (CCPI) assesses international climate policies across four categories: greenhouse gas emissions, renewable energy, energy use, and climate policy [5]. Although no country meets all criteria, the EU ranks high. Southeast Asian nations—Thailand, Vietnam, and Indonesia—perform poorly, and Japan and Malaysia are rated very low. This region’s average temperature has risen by 0.14 °C–0.20 °C per decade since the 1960s, with projections of increased heat and more frequent heatwaves [6].

Education is crucial in addressing climate change challenges, fostering awareness, and promoting sustainable practices. Hence, it is necessary to examine how planetary health is taught—particularly in regions with poor climate performance. Although the importance of PHE is recognized, significant gaps exist in our understanding of its global implementation, especially in environmentally challenged regions. Current knowledge lacks a comprehensive overview of education programs, their effectiveness, and associated challenges. Limited information exists on how cultural, socioeconomic, and political factors influence the integration of planetary health content into existing curricula.

This review focuses on higher education institutions and professional training settings, as these environments are essential for cultivating future leaders who can integrate planetary health principles into policy, education, and clinical practice. PHE requires an advanced understanding of interdisciplinary concepts, making it most applicable at the university level or beyond.

### Objectives

This scoping review aimed to investigate how PHE is implemented across educational settings and to identify current challenges and best practices. This review intentionally focuses on higher education and professional training settings, excluding primary and secondary education, due to the complex and interdisciplinary nature of planetary health concepts. The findings will inform the future development and implementation of planetary health educational programs for university students, educators, and healthcare professionals, who are potential future leaders in promoting societal well-being.

### Research questions

Primary question: What is the current state of PHE implementation across various educational settings?

Secondary questions:

• What are PHE programs’ key components and delivery methods?
• What are the challenges and barriers to implementing PHE?
• What are the outcomes and effectiveness of existing PHE programs?
• How is PHE integrated into existing curricula for students, educators, and healthcare professionals?

The review will map the current literature on PHE to identify evidence gaps and inform future educational program development. This is particularly important, given that the concept of planetary health represents a relatively new educational paradigm that connects human health with the state of natural systems.

## Methods

### Design

This review followed Arksey and O’Malley’s scoping review methodological framework [7] and adhered to the PRISMA-ScR checklist [8]. The research protocol was published in Research Protocols [9].

Scoping reviews map a field of study to identify what is known about a topic and to highlight important evidence gaps that may require further research [7]. This review focused on research about PHE implementation. We used the Patient, Concept, Context (PCC) framework and defined it as follows:

• P (Participants): Students, educators, and healthcare professionals.
• C (Concept): PHE and its delivery methods, educational content, program design, and curriculum.
• C (Context): Geographic location, educational settings, cultural or political background, and specific socioeconomic context.

### Operational definition and conceptual boundary of PHE

For study selection, we operationally defined PHE as structured educational activities (e.g., courses, modules, workshops, curricula, training programs) that explicitly framed their educational aims, content, or competencies as planetary health, and that addressed the interdependence between human health and the integrity of Earth’s natural systems. Consistent with the PHE Framework, eligible studies were expected to include at least one of the following core elements: (i) explicit linkage between environmental change (e.g., climate change, biodiversity loss, pollution, land-use change, resource depletion) and human health outcomes; (ii) systems thinking across ecological and societal determinants of health; (iii) solution-oriented approaches (e.g., mitigation/adaptation with health co-benefits, resilience, policy and governance, community engagement); and/or (iv) equity/justice and transdisciplinary or interprofessional perspectives.

To distinguish PHE from adjacent educational domains (climate change education, sustainability education, environmental health education, and global health education), we applied an additional conceptual screening criterion beyond the presence of the search term. Studies were included when they (a) explicitly used “planetary health” as the primary educational framing (e.g., in the title, abstract, author keywords, learning objectives, curriculum, or course/program name) and (b) described educational content or outcomes that linked environmental change to human health and/or aligned with the above core elements. Studies were excluded if “planetary health” was used only as a general synonym for sustainability or climate change without a clear health-focused framing, or if the described education addressed environmental sustainability without an explicit connection to human health, health systems, or planetary health-related competencies.

### Search strategy

Between May 2024 and September 2025, we searched Web of Science, Cochrane Central Register of Controlled Trials (CENTRAL), CINAHL, Scopus, EMBASE, PubMed, OpenGrey, and Ichushi-Web for English and Japanese literature. We included records published up to September 30, 2025, with no lower limit on publication year. The search strategy was adapted to each database (e.g., field tags and controlled vocabulary). For example, in PubMed we used the following core strategy: ("Planetary health"[Title/Abstract]) AND ("education"[MeSH Terms]). Where available in the retrieved records, we additionally screened the author keywords field for “planetary health” terminology. All references were imported into Rayyan for deduplication and screening. The reference lists of included studies were inspected (backward citation searching) to identify additional relevant studies for inclusion in the review.

### Study selection

**Fig. 1:**
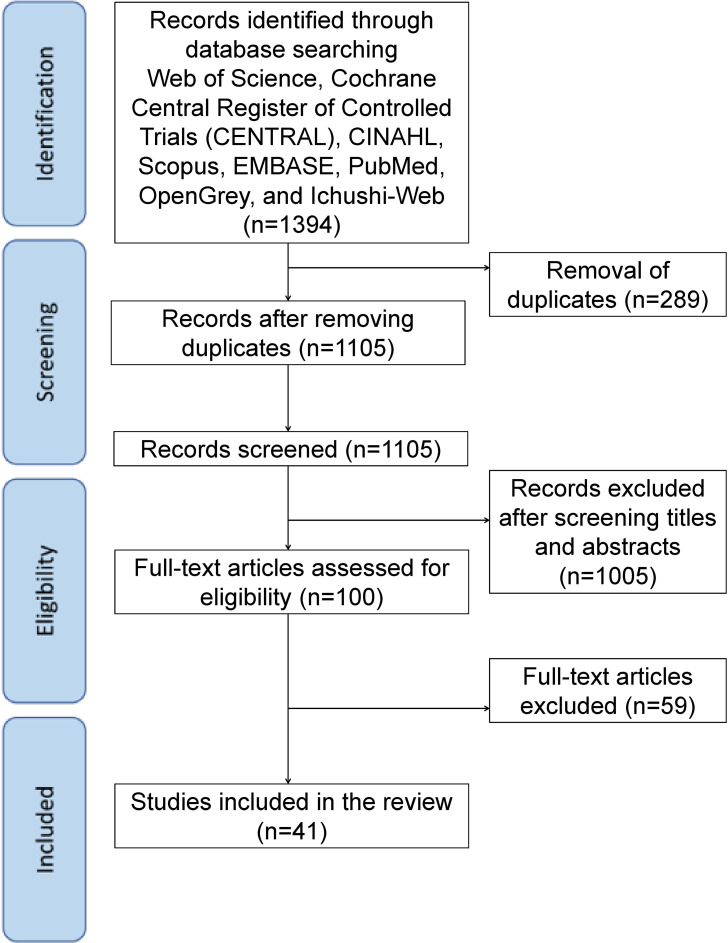
Flowchart of the study selection process. Records were identified through database searches (n = 1,394), duplicates were removed (n = 289), and titles/abstracts were screened (n = 1,105). Full-text articles assessed for eligibility were 100, of which 59 were excluded; 41 studies were included in the review. The study characteristics, methodologies, and key findings of the included studies are summarized in Table 1.

The study selection process was conducted using Rayyan and Microsoft Excel. Two reviewers independently screened records by title and abstract during the initial screening based on the eligibility criteria. Full texts were retrieved and assessed when eligibility could not be determined from the title/abstract alone or when records were identified through backward citation searching. When uncertainty arose, a third researcher was consulted for a definitive decision. During screening, we applied the above operational definition to ensure that included studies had a planetary health framing beyond general climate change or sustainability education. Original journal articles and grey literature discussing PHE were included, whereas letters, comments, books, and reviews were excluded. Conference proceedings were considered eligible; however, none met the inclusion criteria and were therefore not included in the final sample. After deduplication (n = 289), the remaining records (n = 1,105) underwent title/abstract screening. Records were excluded at this stage when they (i) did not focus on education/training, (ii) were not framed as planetary health education according to our operational definition, (iii) were outside the targeted context (higher education or professional training), (iv) were not in English or Japanese, or (v) were publication types excluded a priori (e.g., reviews, books, letters, comments). Full texts were assessed for 100 records; 59 were excluded primarily because they did not meet the operational definition of PHE, did not report an educational program/intervention, or were outside the predefined context or publication/language criteria. The detailed flow is presented in Fig. 1.

### Data extraction

Data extraction was performed using Rayyan and Microsoft Excel. Following Garrard et al.’s matrix method [10], characteristics were organized into eight categories: author(s), publication year, country, objectives, study design, subjects, survey items, and results.

### Analysis

The identified papers were independently reviewed by two reviewers and categorized by research method, target population, and topic. Educational interventions were extracted and described in detail, and country-level indicators (PHDI and CCPI) were summarized and interpreted where relevant. Throughout this process, the review team received guidance from two supervisors experienced in systematic and scoping reviews to ensure consistency and appropriateness. The review was reported in accordance with the PRISMA-ScR checklist.

### Data synthesis

The results of the included studies underwent narrative synthesis using qualitative methods. Where feasible, findings were categorized within the domains proposed by the Planetary Health Education Framework (PHEF) [11]. Common themes across the included studies were identified and analyzed. The synthesis included:

• A description of the study characteristics
• An analysis of educational contexts and settings
• An assessment of program design and implementation methods
• An evaluation of educational outcomes and challenges

The results are presented in tables to compare study characteristics and extracted data, providing a comprehensive overview of the current status of PHE. In addition, to strengthen framework-based analysis, we mapped each included study to the PHEF core elements (i–iv) and provide the detailed mapping in Supplementary Table S1.

### Ethics

As this scoping review involved publicly accessible databases and did not include any interventions or patient recruitment, institutional research ethics committee approval was not required. The findings will be disseminated through peer-reviewed publications and conference presentations to inform the future development of university-level planetary health educational programs.

## Results

### Study selection

A total of 1394 records were initially identified through database searches. After removing duplicates and screening titles and abstracts, 100 articles were selected for full-text review. Subsequently, 41 studies met the eligibility criteria and were included in the final scoping review. The selection process is illustrated in Fig. 1.

### Implementation status and current situation

PHE across diverse regions, including Brazil [12, 13], Australia [14–21], Germany [22–34], the United States [35–42], the Netherlands [43, 44], Canada [45–47], Italy [48], Egypt [49], and Ireland [50, 51], among others. The following results were obtained from an analysis of the HDI and CCPI for these countries. Regarding the HDI, all countries except Brazil (0.786, ranked 84th globally) and Egypt (0.754, ranked 100th globally) are ranked within the top 30 globally [52]. As for the CCPI, the Netherlands (5th), Egypt (20th), Ireland (29th), Germany (16th) and Brazil (28th) are within the top 30. In contrast, Italy (43rd), Australia (52nd), Canada (62nd), and the United States (57th) are ranked lower [53].

At the educational level, implementation was predominantly observed in higher education institutions, particularly medical [12–17, 22–28, 30–35, 37, 39, 44, 45, 48, 49] and nursing [19–21, 41, 46] schools. Recent initiatives demonstrate wider disciplinary coverage—for example, the integration of a blended-learning module titled “Child Health in Times of Planetary Crises” into pediatrics curricula [32], nationwide PHE implementation across 90% of German medical schools [33], and cross-national student-led curriculum reform projects in the United States [40]. In Italy, an elective course for fifth-year medical students yielded strong motivation and satisfaction related to the climate crisis [48], while in Egypt, an online elective course on planetary medicine for 206 students significantly enhanced knowledge and satisfaction [49]. In the nursing context, national audits across Australia, New Zealand, and Canada revealed limited curricular integration, often confined to elective components or single modules [20, 46]. Rosenau et al. [29] introduced education through practical cooking sessions for university students.

Although public universities were the primary sector, some programs, such as the one in the study by Floss et al. [12]—were open to the general public through Massive Open Online Course (MOOC) formats. The Canadian TRASH-CAN initiative [47] demonstrated the feasibility of national, trainee-driven Quality Improvement (QI) projects promoting sustainable practice through web-based collaboration.

### Educational program components and implementation methods

Primary themes included climate change and health [17, 22, 23, 28, 36–39, 41, 45], environmental hygiene [15, 54], public health [14, 24, 35], and the Sustainable Development Goals (SDGs) [16, 29, 43]. Although climate change and health was the most frequently reported theme, several programs also addressed other Earth-system changes and related health pathways, such as pollution and waste reduction, food systems and sustainable diets, and environmental hygiene (Table 1).

**Table 1:** Overview of Included Studies on Planetary Health Education

**No**	**In-text** **citation**	**Authors** **(year)**	**Country**	**Objective**	**Research Design**	**Participants**	**Survey Items**	**Educational** **Intervention**	**Results**
1	13	Zandavalli RB et al. (2024)	Brazil	To evaluate Brazilian medical students’ perceptions of planetary health and assess an educational material using Actor-Network Theory.	Mixed-methods, quasi-experimental design	96 medical students (3rd semester) and 9 professors	1. Pre- and post-intervention closed-ended questions on perceptions related to PH 2. Open-ended questions on experiences and learning 3. Sociodemographic data	PH module integrated into a medical course, with patient interviews and portfolio creation emphasizing epidemiological, clinical, and systems perspectives. Activities included a contemplative trail and planetary belonging reflections.	66.7% of students reported significant learning. PH-related disease knowledge improved (p < 0.02). Increased sense of environmental connection (p = 0.003) and PH integration in practice (p = 0.002). 83% found sessions relevant.
2	14	Christian M et al. (2023)	Australia	To integrate planetary health into a medical preparation program to enhance student awareness and understanding.	Case study with qualitative and quantitative methods	100 first-year students enrolled in a 12-week Physiology subject at an Australian university, including biomedical science, health science, exercise science, and pre-medical pathway students	1. Students’ knowledge and attitudes towards planetary health	Planetary health integrated into Physiology curriculum through lectures, group discussions, and hands-on workshops to teach students about planetary health’s importance.	Students’ knowledge and awareness of planetary health significantly improved after the program.
3†	22	Weber A et al. (2023)	Germany	To investigate students’ awareness of climate change health impacts and their engagement in sustainable behaviors.	Cross-sectional study	3756 students from the University of Regensburg, Germany (out of 20,678 total students)	1. Demographic data 2. Eating habits 3. Alcohol consumption, psychoactive substances, smoking 4. Physical activity and health promotion 5. Psychological and physical impairments 6. Academic and welfare 7. Global health and sustainable behavior 8. Hopes for the future of the university campus	Universities should promote sustainable choices by providing sustainable infrastructure, healthy food options, and educational programs raising awareness of planetary health and sustainable development.	Medical and natural sciences students had higher climate-health knowledge than law, economics, and computer science students. Higher knowledge correlated with sustainable behaviors. Full vegan adoption intent was low.
4†	23	Fülbert H et al. (2023)	Germany	To introduce “Climate-Sensitive Health Counseling” to improve medical students’ understanding of climate change and health.	Participatory, student-led elective course with interactive seminars and practical exercises	A total of 25 students from the University of Giessen and the University of Marburg, Germany, participated in this elective course (15 students from the University of Giessen and 10 students from the University of Marburg).	1. Interaction between climate change and health 2. Transformative action and health behaviors 3. “Green hospital” concept 4. Climate-sensitive health counseling simulations 5. Student feedback on the course, particularly regarding the need for more education on climate change and health in medical curricula	Nine-session climate-health course included theoretical knowledge, practical training, climate-sensitive counseling, green hospital concepts, and behavior changes promoting sustainability in healthcare.	The elective course was well-received, confirming high demand for climate-health education. Adaptability across two universities was demonstrated, and many students planned to apply acquired knowledge in clinical practice.
5†	35	Alonso Luaces M et al. (2021)	USA	To describe and evaluate a virtual global health elective curriculum.	Pre-post self-assessment with qualitative thematic analysis	82 trainees enrolled in the virtual global health elective from January to May 2021	1. Self-assessed competency improvement in global health, planetary health, low-resource clinical reasoning, and overall composite competency 2. Qualitative responses on learner development in health systems, social determinants of health, critical thinking, cultural humility, and professional practice	Virtual global health elective with faculty from multiple countries, enhancing global health competencies and participant diversity.	Self-reported competencies in global health, planetary health, and low-resource clinical reasoning significantly increased. 40% of participants were from outside the U.S.
6†	24	Dumm M et al. (2023)	Germany	To assess nutrition education in German medical curricula and the impact of a student-led online program.	Survey study	Over 1500 medical students from German medical faculties	1. Self-assessed nutrition competence and knowledge 2. Attitudes towards nutrition	“Eat This!” online student- led program with 11 digital lectures improving students’ nutrition-related competencies.	Insufficient nutrition education contributed to physicians’ lack of confidence in counseling. A student-led online program significantly improved self-perceived competence, knowledge, and attitudes.
7†	15	Huang A et al. (2024)	Australia	To understand Australian healthcare staff’s perceptions of sustainable healthcare and necessary organizational support.	Descriptive cross-sectional survey with quantitative and qualitative data	301 staff members from a large hospital and healthcare service organization in northeastern Queensland, Australia, including Sunshine Coast Hospital and Health Service	1. Attitudes towards environmentally sustainable and climate-resilient healthcare	Proposed strategies for climate-resilient healthcare, including sustainability training, system integration, and practical staff support.	Staff strongly support sustainable, climate-resilient healthcare but feel there is insufficient organizational support.
8	16	McLean M et al. (2022)	Australia	To evaluate a learner-centered planetary health assignment in a medical program at Bond University.	Evaluation research on a team-based, learner-centered planetary health assignment	162 teams of 4–6 students (spanning 2018–2022) from the second year of the five-year medical program at Bond University	1. Planetary health problems chosen by the teams 2. Selected SDGs 3. Intended audience for proposed solutions 4. Self-assessment of personal and professional development	Students engaged in global issues, proposing solutions, drafting policy proposals, and improving awareness, digital skills, problem-solving, and teamwork.	Key concerns: waste (35.4%), pollution (31.1%), and health deterioration (19.3%). Top SDGs: SDG12 (41%), SDG3 (22%), and SDG6 (15%).
9	17	Burch H et al. (2023)	Australia	To develop a medical curriculum integrating planetary health and climate change impacts.	Stepwise approach with literature review, student focus groups, workshops, and expert review	26 medical students (workshop participants)	1. Climate change-related teaching opportunities and students’ perceptions	Two five-hour workshops where students and faculty co-developed an open- access planetary health curriculum resource mapped to organ systems teaching.	Through student focus groups, workshops, and expert review, a comprehensive infographic linking planetary health to organ systems was developed.
10†	54	Duane B et al. (2024)	Multinational (Ireland, UK, Colombia, Portugal, Spain, Germany, Chile, Italy, Belgium)	To explore integrating environmental sustainability into dental curricula with practical guidelines.	Scoping review with qualitative workshop discussions	Participants of the ADEE sustainability workshop and related experts (No specific number of people listed)	1. Understanding of environmental sustainability in dental education	Environmental sustainability integrated into dental education through educational materials and learning outcomes.	Workshop discussions focused on disease prevention, patient education, lean service delivery, and environmentally sustainable strategies.
11	50	Nordrum OL et al. (2022)	Ireland	To assess planetary health education in Irish medical schools, including integration and implementation.	Cross-sectional survey using the Planetary Health Report Card initiative	Student representatives from each of the five Irish medical schools (UCD, TCD, NUIG, UCC, and RCSI) participated in the evaluation. The majority of the participating student researchers were in their middle or later years of medical training (No specific number of people listed)	1. Presence of learning outcomes and topics related to planetary health in the curriculum 2. Collaboration between research centers and medical schools	Medical schools must integrate planetary health systematically, enhance collaboration with research centers, and ensure graduates are prepared as medical leaders in planetary health.	Few Irish medical curricula explicitly address planetary health. Integration depends on individual lecturers. Strong PH research centers exist but are poorly connected to curricula.
12	12	Floss S et al. (2021)	Brazil	To design and evaluate a Massive Open Online Course (MOOC) on planetary health for professionals and the public.	MOOC development with course design, content creation, and evaluation methods	2777 people enrolled, with 1237 participants giving informed consent; majority were women under 35, concentrated in southern and southeastern Brazil, with some participants from Mozambique, Chile, and Peru	1. Participant profile and demographic data 2. Pre-course knowledge and post-module tests 3. Final evaluation and action plan submission 4. Surveys and forum activities	MOOC combined scientific rigor with emotional and creative elements, hosted on Moodle, accessible in low-bandwidth environments.	Many students lacked PH knowledge but recognized its importance. Key topics in action plans: food and nutrition, infectious diseases, and waste/recycling.
13	25	Simon J et al. (2023)	Germany	To examine high-quality planetary health education from stakeholders’ perspectives and compare with existing frameworks.	Qualitative interview study	A total of 20 participants (13 female) from 15 different medical schools were interviewed. The participants were stakeholders involved in Planetary Health Education (PHE), including faculty members, medical students actively involved in PHE, and study deans of medical schools.	1. Characteristics of high-quality PHE (complexity, systems thinking, interdisciplinarity, ethics, responsibility of healthcare professionals, transformative competencies, reflection space, and the role of students)	Study explored existing planetary health education (PHE) practices rather than implementing an intervention but identified ten characteristics to guide future curriculum development.	A qualitative study explored the perspectives of stakeholders at German medical schools on the characteristics of high-quality planetary health education (PHE), identifying ten key themes that can be useful for designing and implementing new educational activities.
14	43	Dambre et al. (2022)	The Netherlands	To evaluate interdisciplinary and transcultural approaches in planetary health education at the University of Groningen.	Focus group interviews	8 students from the Bachelor’s program in Global Responsibility & Leadership at the University of Groningen, majoring in Social Sciences, Environmental Studies, and Governance	1. Student feedback on interdisciplinary and peer-to-peer learning approaches	Nine-week planetary health elective featuring interdisciplinary expert knowledge, peer-to-peer learning, and student-led sessions encouraging deeper exploration of specific topics.	Students valued transdisciplinary learning, appreciating its slower, inclusive approach. A mix of vertical (expert-led) and horizontal (peer-to-peer) learning was most effective.
15	41	Cygan H et al. (2024)	USA	To understand nursing students’ and faculty opinions on climate change and planetary health curriculum needs.	Pilot study with quantitative research methods	72 nursing students and 56 faculty members at Rush University College of Nursing	1. Demographic data of participants 2. Personal opinions on climate change	Nursing curriculum should integrate climate-health education through in-service and extracurricular activities, as well as core course sessions.	Students and faculty were concerned about climate change but felt nursing education was unprepared to address its impacts.
16	26	Klünder et al. (2023)	Germany	To assess students’ understanding of planetary health and interest in elective courses in Bavarian universities.	Cross-sectional study	1479 students enrolled in self-defined health-related fields in Bavaria (1303 responses)	1. Prior knowledge of Planetary Health 2. Interest in learning about Planetary Health 3. Willingness to participate in a Planetary Health elective	Survey-based strategy proposed for interdisciplinary online electives, developing planetary health integration into health-related degree programs.	73.8% were unfamiliar with planetary health, but 90.7% expressed interest in learning. 81.9% would join an online elective.
17	45	Luo OD et al. (2023)	Canada	To incorporate “Climate Wise” slides into medical curricula to improve planetary health knowledge and physician training.	Pre-post comparison study	75 Canadian medical students	1. Planetary health knowledge score (before and after lecture) 2. Interest in including “Climate Wise” slides in the curriculum	“Climate Wise” virtual lecture session with pre/ post-lecture questionnaires using an evidence-based pedagogical tool.	Significant improvement in planetary health knowledge scores (p < 0.0001). Increased interest in including slides in the curriculum (p < 0.001).
18	27	Schmid J et al. (2023)	Germany	To introduce a longitudinal planetary health curriculum with a learning spiral approach.	Project implementation and evaluation	The study involved a physician and student assistant, who implemented the project, alongside teaching staff and course coordinators from 26 different specialities at the Faculty of Medicine in Würzburg. Local medical students also participated.	1. Comparison of existing curriculum learning objectives on planetary health 2. Interest in integrating planetary health topics into the curriculum	Online event series for faculty and students, regular faculty meetings, new planetary health lectures in environmental medicine, and evaluation via student/faculty surveys.	PH topics were integrated into multiple lectures with structured learning objectives in knowledge, attitudes, skills, and confidence.
19†	36	Shea K et al. (2020)	USA	To analyze the state of climate-health curricula in health professions institutions globally.	Survey research	160 institutions from the Global Consortium on Climate and Health Education (GCCHE)	1. Climate-health education offerings and curriculum content (required vs. elective) 2. Consideration of adding climate-health education 3. Responses to curriculum addition (students, faculty, administration) 4. Challenges and opportunities for implementation	No specific intervention but insights provided to guide climate-health curriculum development.	63% of institutions offer climate-health education, mostly in core courses. 74% are considering it. Key barriers: staff time (71%) and funding (34%).
20†	37	Liu I et al. (2022)	USA	To evaluate medical students’ perspectives on a climate change and health curriculum.	Focus group interviews	13 randomly selected students from a cohort of 139 students scheduled to graduate in 2024 from Emory University School of Medicine	1. Pre-entrance awareness of climate change and health 2. Current thoughts on climate change and health in a medical career 3. Feedback on the existing curriculum and suggestions for improvement	Student co-created curriculum integrating climate-health topics into existing courses using participatory, small group, and case-based approaches.	Students valued integrating climate-health content into medical curricula. Participatory learning (group discussions, case-based learning) was preferred over traditional lectures.
21	51	Bates OB et al. (2022)	Ireland	To investigate factors influencing planetary health integration in Royal College of Surgeons in Ireland’s medical curriculum.	Qualitative descriptive study with semi-structured interviews.	12 academic staff members actively involved in teaching in the undergraduate medical curriculum at the Royal College of Surgeons in Ireland University of Medicine and Health Sciences	1. Awareness and perceptions of climate change and health topics 2. Knowledge and attitudes towards climate change 3. Personal and professional actions for climate change mitigation 4. Course evaluation	Curriculum recommendations included integrating planetary health with clinical relevance, leadership promotion, and institutional prestige enhancement.	Barriers: lack of curricular space, low awareness, and knowledge gaps. Facilitators: growing student demand, innovative teaching, and clinical relevance emphasis.
22†	38	Blanchard OA et al. (2022)	USA	To identify barriers and facilitators to integrating climate change and health into U.S. medical curricula through interviews.	Qualitative descriptive study with semi-structured interviews	9 faculty members involved in medical education at medical schools in the United States	1. Awareness and perceptions of climate change and health topics	Global need to integrate planetary health into medical curricula, requiring substantial curricular revisions.	Barriers: lack of curricular space, low awareness, and knowledge gaps. Mitigators: growing student and faculty demand, innovative content delivery, and emphasis on clinical relevance.
23†	28	Lemke D et al. (2022)	Germany	To introduce and evaluate the first climate change and health elective at Goethe University Frankfurt.	Qualitative descriptive study with pre- and post-course questionnaire surveys	18 students enrolled in the clinical program at Goethe University Frankfurt, including 4 students from non-medical fields	1. Knowledge of climate change and health 2. Attitudes towards climate change 3. Personal actions for climate change mitigation 4. Professional actions as future physicians 5. Course evaluation	“Climate Change and Health” elective course included live online sessions, preparatory learning, discussions, case studies, and a reflection assignment.	Students reported high satisfaction. Knowledge, attitudes, and behaviors on climate-health significantly improved. Most advocated for curriculum integration.
24	29	Rosenau N et al. (2023)	Germany	To implement and assess a planetary health diet curriculum in a teaching kitchen setting.	Prospective intervention study with pre- and post-course surveys	26 university students from various degree programs at the University of Göttingen	1. Pre- and post-survey on self-assessed planetary health diet literacy 2. Course evaluation questionnaire based on the University of Göttingen’s official teaching evaluation	Seven flipped classroom sessions with one-hour seminars, student presentations, and two-hour hands-on cooking classes on nutrition and sustainability.	25 students provided evaluations, reporting high satisfaction. Self-assessed planetary health diet literacy improved by 21–98%.
25	30	Teichgräber U et al. (2024)	Germany	To evaluate OSCE as an educational tool for planetary healthcare training.	Observational study with an elective planetary health course and OSCE evaluation	20 senior medical students (not all participated in the elective course)	1. Survey using a nine-point Likert scale to assess the usefulness of each OSCE scenario and its integration into the curriculum 2. Group interview on incorporating planetary health care and management into the curriculum and students’ roles 3. OSCE performance assessment by examiners and peer feedback	OSCE enhanced professional competencies, integrating knowledge and communication skills, raising awareness, and empowering others in health and environmental goals.	OSCE scenarios were seen as highly relevant. Peer feedback was valued. Examiner assessments highlighted communication challenges, especially with colleagues and administrators.
26	31	Schwienhorst-Stich EM et al. (2023)	Germany	To analyze planetary health education’s impact on students’ emotions and climate action motivation.	Pre-test/post-test design with anonymous online surveys	458 medical students (349 students in Environmental Medicine lectures, 109 in the optional planetary health course); 396 students participated in the final survey (288 from lectures, 108 from the optional course)	1. 20 closed-ended questions using a 5-point Likert scale 2. 6 open-ended questions about students’ emotions and motivations related to climate change	One-term planetary health elective (40 units) plus Environmental Medicine lectures on action options and emotional resilience.	Initial emotions: helplessness (57%), fear (51%). Post-lecture changes: reduced helplessness (−0.37) and disappointment (−0.35), increased confidence (+0.67) and motivation to act (+0.4).
27†	39	Ramkumar J et al. (2021)	USA	To develop and evaluate a standardized patient case on asthma exacerbation due to wildfire smoke.	Simulation-based study with a standardized patient case and post-simulation assessments	11 internal medicine clerkship students (third-year medical students)	1. Students’ awareness, knowledge, and attitudes toward climate change and its health impacts 2. Post-simulation comments and learner assessments	Standardized patient (SP) simulation on wildfire smoke and asthma, part of OSCE, assessing clinical and communication skills, followed by faculty debriefing.	Improved awareness, knowledge, and attitudes on climate-health (p = 0.006). Post-simulation, students better considered climate-exacerbated health risks.
28	18	Hickman AC et al. (2022)	Australia	To explore reflexivity’s role in health promotion education for planetary health learning and behavior change.	Brief report on course redesign with reflexive educational methods and student feedback evaluation	Students enrolled in a postgraduate health promotion course (PUBH7034) (No specific number of people listed)	1. Student feedback on reflexive teaching methods and related challenges 2. Student engagement with reflexive assignments and reflections on their learning experience	Reflexivity framework included reflective blogs, research reports, and advocacy actions, with feedback loops supporting student learning.	Students responded positively to reflective learning. Many took action by sending advocacy letters. The nested assignment design effectively built confidence.
29	32	Block S. et al. (2025)	Germany	To co-developa student-driven, interactive, competence-based pediatric PHE module on climate-sensitive health counselling (CSHC) and evaluate medical students’ pediatric planetary health literacy.	Mixed methods study	67 medical students were included in the final analysis (response rate 90.54%). All participants were in their tenth semester of medical studies. 74 students participated in the entire module.	The study utilized two questionnaires (pre- and post-course): 1. Demographic characteristics. 2. Planetary Health Literacy assessment: 11 statements. 3. Module didactic design evaluation: 7 statements on a 5-point Likert scale. 4. Three open-ended questions for free-text answers regarding the learning experience (Post-evaluation only).	A mandatory blended learning module titled “Child Health in Times of Planetary Crises”. It was integrated into the pediatrics curriculum. The approach was student-led and used a peer-teaching model. The module components were: an Online course, a Seminar, and role-playing. The module was developed based on expert interviews with pediatricians practicing CSHC.	Students’ planetary health literacy improved significantly across knowledge, comprehension, and self-assessed competence to apply CSHC (p < 0.01).
30	33	Grieco F. et al. (2025)	Germany	To assess the current implementation status of Planetary Health Education (PHE) in undergraduate medical education in Germany, and to explore its characteristics	Mixed-methods, sequential design.	All 39 German medical schools were targeted. Interviews: 80% participation (50 individuals including students, educators, and deanery staff). Online Survey: 90% participation (66 individuals met inclusion criteria, mainly educators and deanery staff).	Questionnaires covered general information, learning objectives (LOs), teaching and assessment methods, cooperation (interdisciplinarity, student involvement), and faculty support for PH. Inclusion required covering foundational knowledge Los.	The study assessed existing PHE activities (stand-alone courses or integrated lectures). Mandatory activities predominantly used lectures and Multiple Choice Quizzes (MCQ). Electives used more varied, innovative approaches (e.g., simulation, small group work) and targeted upper competency levels.	90% of medical schools offered PHE (median of two activities). The majority (66%–69%) were electives and not mandatory curriculum. High-quality PHE criteria (innovative approaches, transformative learning) were primarily reflected in electives. LOs related to transformative competencies were significantly more emphasized in electives (94% vs. 71%; p = 0.0176). Student involvement was significantly associated with transformative learning objectives (p = 0.002). Mandatory PHE remains insufficiently integrated.
31	40	Malani, K et al. (2025)	USA	To evaluate challenges and successes faced by medical students who are leading Planetary Health (PH) curriculum reform and to track changes over time. The study also aimed to identify strategies to increase PH integration at various levels.	A mixed-methods approach was used, incorporating surveys, qualitative interviews, and analysis of publicly available Planetary Health Report Card (PHRC) data.	31 students from 17 US states completed the survey, selected from 132 eligible primary PHRC contacts across 52 US institutions. Interview Respondents: 11 students in their third or fourth year of medical school completed one-on-one Zoom interviews. PHRC Data Analysis: 32 US medical schools with more than one PHRC report card	Surveys assessed challenges, successes, stakeholders, stages, resources, and barriers (Likert scale). Interviews examined current PH curriculum integration, stakeholders, challenges, resources, and ideal inclusion level.	This study is an analysis of student-led curriculum reform efforts and their challenges and successes, rather than the assessment of a specific educational intervention. The research recommends that medical schools prioritize developing longitudinal, course-specific PH education, supported by individual institutions and national frameworks and licensing organizations, to better equip future healthcare professionals.	Students are key drivers of PH curricular reform, but only 9.7% perceived instructors as confident in teaching PH. PH is often delivered as a standalone course, and all interviewees reported insufficient integration. Key barriers included limited curricular space, unclear national accountability, and limited faculty capacity. PHRC scores increased from 2020 to 2023.
32	34	Charlotte Flock et al. (2025)	Germany	To explore Final Year Medical Students (FYMS)’ perspectives on integrating Planetary Health Education (PHE) into medical curricula and their perceived relevance and o responsibility regarding climate change/health in their future.	Qualitative interview study.	10 volunteering Final Year Medical Students (FYMS) from the Department of Internal Medicine at Heidelberg University Hospital	A semi-structured interview guide was used. Key areas explored included: students’ views on the role of climate change in their future profession, preferences for integration into the curriculum (knowledge, skills, attitudes), and perceived professional responsibility to address climate change and health with patients.	The study itself did not implement an educational intervention. It was conducted *independently* of a planned informational event (a themed week) for FYMS, serving the broader purpose of gathering expectations for *future* curriculum development.	FYMS recognized the relevance of climate change to their future practice but showed varying degrees of perceived responsibility in addressing it with patients, often depending on their desired specialization. Students strongly wished for content on health impacts of climate change, communication skills (especially addressing skeptics), and interactive, practice-oriented teaching methods (e.g., case work). Reservations were noted concerning the integration of basic climate science principles and the introduction of mandatory standalone exams.
33	48	L. Nachira et al. (2025)	Italy	To assess medical students’ expectations and feedback regarding a Planetary Health (PH) elective course and evaluate the relevance of integrating PHE into the medical curriculum	Cross-sectional study utilizing a convergent parallel mixed-methods design.	Fifth-year medical students at UCSC. 80 enrolled; 74 completed pre-course; 33 completed post-course.	Three anonymous questionnaires administered: Pre-course, Post-Educational Sessions (n = 4), and Post-course.	A PH elective course at UCSC consisting of four 2-hour sessions covering general PH issues.	Main motivation was interest in the climate crisis (52.7%). Five qualitative themes emerged, including the relationship between human health and the environment. Satisfaction was high, and students showed a deep understanding of PH relevance.
34	49	Hanaa Saeed Elhoshy et al. (2025)	Egypt	To evaluate the effectiveness of an online elective course on sustainable healthcare and planetary medicine for undergraduate medical students. Specific aims included assessing baseline knowledge, enhancing knowledge acquisition (Level 2), gauging satisfaction (Level 1), and identifying improvements.	Quasi-experimental study using a one-group pretest–posttest design. Evaluation focused on Kirkpatrick’s Level 1 (Reaction) and Level 2 (Learning).	A sample of 206 third-year medical students from Alexandria University. This sample represented 7.99% of the total third-year population.	Level 2 (Learning): 20-item identical Multiple-Choice Questions (MCQs) for pretest/posttest. Level 1 (Reaction): 14-item online satisfaction questionnaire (5-point Likert scale). The pretest also assessed student attitudes and perceptions.	An online elective course on planetary health and sustainable healthcare lasting 12 days, with a total workload of 15–20 hours. Course development was guided by the Community of Inquiry framework. Utilized Moodle, Zoom, and WhatsApp for asynchronous lectures and synchronous sessions. Included group research projects (infographics, concept maps).	Statistically significant improvement in knowledge was confirmed: posttest scores (M = 16.69) were significantly higher than pretest scores (M = 12.41) (p < 0.05). Student satisfaction was high, with the overall course rating being the highest item (M = 4.4 ± 0.70). Before the course, 93.2% of students expressed a need for more education in this area.
35†	19	Wang et al. (2025)	Australia	To explore the impact of an education module embedding sustainability principles in clinical simulation. The aim was to measure the impact on student attitudes, knowledge, and practices relating to climate change and sustainability.	Mixed methods study using pre- and post-intervention waste audits and student surveys.	173 second-year undergraduate nursing students at an Australian university. 44 (30.3%) pre-survey and 80 (53.3%) post-survey completed.	Adapted SANS_2 tool (items 1–5). Added items (6, 7) on sustainability application in clinical simulation and practice, and items (8–10) on perceived frequency of applying ‘reduce,’ ‘re-use,’ and ‘recycle’. Open-ended questions gathered qualitative data on facilitators and barriers.	A digital clinical simulation education module was embedded in an existing unit. Key themes included 1) professional conduct, 2) work health and safety, and 3) resource stewardship and sustainability.	Quantitative: No significant difference in general or sharps waste per student was detected post-intervention. The perceived application of sustainability during clinical simulation significantly increased (mean 4.8 to 5.4, p ≤ 0.01). Perceived application of ‘reduce’ and ‘re-use’ principles also significantly increased. Qualitative (Barriers): Barriers included cognitive overload, knowledge deficit, time pressure, and a desire for authentic practice.
36	20	Tutticci, N et al. (2025)	Australia and New Zealand	To obtain a snapshot of planetary health theory and practice in nursing curricula to inform future education development at professional and policy levels.	Descriptive study using a combination of a web-based desktop audit of curricula and a mixed-method online cross-sectional survey.	Audit: 36 preregistration Bachelor of Nursing (BN) programs in Australia and New Zealand. Survey: Program directors/faculty directors (4 out of 36 invited universities responded, 10% rate).	Audit: Included terms like sustainability, planetary health, climate change, environmental determinants of health, and emissions reduction in course descriptions, learning objectives, or outcomes (Q1 & Q2). Survey: Program directors’ perceived importance of climate change and carbon emission reduction in curricula (Q3), including barriers and enablers.	None implemented. However, the study concludes by recommending the pedagogic recontextualization of planetary health using case study concept nodes within a person-centered care framework.	Audit: Only 4 (11%) of 36 BN programs included climate change/health-related terms. Key terms found were Sustainability (n = 2), environmental determinants of health (n = 2), and climate change (n = 2). Survey: The low response rate (10%) supported evidence of limited integration. Two of four respondents indicated inclusion, but barriers like curriculum crowding and lack of regulatory requirements were cited.
37	21	Levett-Jones et al. (2025)	Australia	To investigate the impact of the ‘Healthy Planet, Healthy People’ (HPHP) educational board game on nursing students’ planetary health attitudes, knowledge levels, and satisfaction with the learning experience.	Multicentre pre-post survey design. Wilcoxon Signed Rank Tests were used to assess changes.	184 students from five Australian universities completed both the pre- and post-surveys.	A pre-post survey used three subscales: 1) Knowledge (10-question multiple-choice quiz), 2) Attitudes (7-item 5-point Likert scale), and 3) Satisfaction (12-item 5-point Likert scale plus one open-ended question).	The HPHP educational board game. It takes approximately 45 minutes to play and was conducted in randomly assigned teams. The game focuses on environmental health and the impact of the environment on human health.	There was a statistically significant increase in both attitude (median score increased from 3.86 to 4.00; p < .001, medium effect size r = 0.39) and knowledge levels (median score increased from 5.00 to 7.00; p < .001, large effect size r = 0.73) following the game. Participants reported high levels of satisfaction with the learning experience (25th percentile scores consistently at 4.00). There were no significant differences in attitude or knowledge based on demographic characteristics like gender or employment status in healthcare.
38†	44	Stevens, M. et al. (2024)	The Netherlands	To evaluate medical students’ knowledge and attitudes towards climate change and health following a novel serious game.	Mixed-methods study design. Quantitative data via pre- and post-intervention surveys. Qualitative data via post-intervention focus group discussions.	Third-year bachelor medical students at Erasmus MC. (N = 59 completed pre-/post-surveys; N = 11 participated in focus groups). Participants had no prior formal education on planetary health/climate change and health.	3 parts: (1) Demographics, (2) Knowledge (3 self-reported, 2 objective exam questions), (3) Attitudes towards climate change and health/importance for doctors. Majority utilized 5-point Likert scales.	A novel collaborative serious game (2-hour session) embedded in a public health course. Components included: 1. Card arrangement activity exploring complex interlinkages. 2. Group discussion/reflection on emotions, vulnerabilities, and personal/professional responsibilities (including co-benefits).	Knowledge: Self-reported knowledge significantly increased (p < 0.001). Objective knowledge regarding vulnerability/inequity significantly increased (p < 0.001). Attitudes: Climate worry significantly increased (42.4% to 69.5%; p < 0.001). Significant increase in recognition of future doctors’ role in informing patients (p = 0.003) and society (p = 0.003). Qualitative: Enhanced understanding of complex interlinkages and realization that co-benefits provide tools for action.
39	42	Sarah Schear et al. (2024)	United States, Canada United Kingdom	To adapt the Planetary Health Report Card (PHRC) for Graduate Medical Education (GME) contexts and report preliminary validity evidence.	Modified Delphi Panel method	20 participants (44% of those recruited) completed the first-round survey. Roles included a senior medical student, residents, fellows, faculty, and program directors. Participants had expertise in planetary health, sustainability, and health equity.	Adapted metrics across 5 domains of GME evaluation: (1) Curriculum, (2) Research, (3) Community Outreach, (4) Support for Trainee-Led Initiatives, and (5) Sustainability. Participants scored metrics using a 4-point modified Likert scale.	Not an intervention study. The study developed an evaluation tool (GME PHRC) to assess whether GME programs address topics like climate change and health risks, environmental toxins, health inequity, healthcare carbon footprint, and sustainable healthcare methods.	Trainees and faculty agreed that the adapted metrics are relevant for evaluating GME programs on planetary health, sustainability, and environmental justice. High level of agreement (75% to 100%) was found for all metrics in Curriculum, Support for Trainee-Led Initiatives, and Sustainability domains. Some metrics in Research and Community Outreach domains fell below the agreement threshold (>70%).
40	46	Astle, Barbara, et al. (2025)	Canada	To better understand the integration of Planetary Health (PH) into nursing curricula and pedagogical practices across Canada. To assess the current integration, facilitators, and barriers for PH in Canadian nursing education, and explore the uptake of the PH Education Framework (PHEF).	Online, descriptive survey	Leaders (Deans, Directors, Chairs) at Canadian schools of nursing. Phase 1 included 16 different Anglophone institutions. Institutions were located in seven different Canadian provinces.	A total of 19 questions. Questions aimed to collect information on the type of programs offered, whether PH concepts were (or planned to be) integrated, barriers and facilitators for integration, and knowledge and use of the PHEF. PH was defined by a list of concepts. Items included drop-boxes, a 5-point Likert scale, and comment responses.	Most schools addressed PH concepts through content in core nursing courses (50%, n = 7/14). Fewer reported using a dedicated course (21%, n = 3/14). Common resources used included case studies (n = 6, 43%) and readings/videos (n = 6, 43%).	All responding schools (n = 16) reported addressing one or more PH-related concepts. The most commonly reported concept was “Indigenous land-informed ways of knowing” (n = 13, 81%). All respondents (n = 16) reported plans (or possible plans) to integrate new/additional PH concepts. Major Facilitators: Institutional strategic direction (n = 13) and faculty interest (n = 12). Major Barriers: Already overloaded curriculum (n = 13) and lack of faculty expertise in how to integrate PH (n = 11). Half (50%, n = 8) were aware of the PHEF.
41	47	Best G.M. et al. (2024)	Canada	To reduce Canadian health care’s environmental impact via trainee-led Quality Improvement (QI) To educate and empower trainees to implement sustainable practices and engage in planetary health work.	National, resident-driven initiative (TRASH-CAN). Based on a web-based platform supporting three pillars: Learning, Leadership, and Delivery. Focus on launching and supporting QI projects.	31 enrolled participants (15 faculty mentors and 16 residents/medical students). Participants are from institutions across Canada and a broad range of clinical specialities.	Use of trainee and mentor intake forms. Future plans include surveying the membership’s baseline understanding of sustainable practice in perioperative and inpatient care.	3-Pillar structure: Learning (developing curriculum/promoting literature), Leadership (empowering trainees as sustainability champions), and Delivery (hands-on execution of QI projects focused on waste reduction).	In its first year of operation, TRASH-CAN has developed a fully functional website hosting intake forms and detailing ongoing projects and opportunities. We have enrolled 15 faculty mentors and 16 residents and medical students, with ongoing projects such as transitioning hospitals to reusable alternatives and optimizing procedural custom operating room equipment packs.

Note: All included studies used “planetary health” terminology in at least one record field (title, abstract, author keywords, and/or course/program description). Studies primarily addressing climate change, sustainability, or environmental sustainability were retained only when they aligned with the operational definition (i.e., linking environmental change to human health and/or core elements of the Planetary Health Education Framework). † Studies marked with a dagger did not explicitly use “planetary health” as the primary framing in the title/abstract; they were included because they met the operational definition and used “planetary health” terminology as described above.

Abbreviations: ADEE, Association for Dental Education in Europe; BN, Bachelor of Nursing; CCPI, Climate Change Performance Index; CSHC, climate-sensitive health counselling; FYMS, final-year medical students; GCCHE, Global Consortium on Climate and Health Education; GME, graduate medical education; HPHP, Healthy Planet, Healthy People; MCQ, multiple-choice question; MOOC, massive open online course; OSCE, objective structured clinical examination; PH, planetary health; PHDI, Planetary pressures-adjusted Human Development Index; PHE, planetary health education; PHEF, Planetary Health Education Framework; PHRC, Planetary Health Report Card; QI, quality improvement; SDGs, Sustainable Development Goals; SP, standardized patient; SANS_2, Sustainability Attitudes in Nursing Survey-2; UCD, University College Dublin; TCD, Trinity College Dublin; NUIG, National University of Ireland Galway; UCC, University College Cork; RCSI, Royal College of Surgeons in Ireland; UCSC, Università Cattolica del Sacro Cuore; USA, United States of America; UK, United Kingdom.

To facilitate interpretation, we added aggregate tables summarizing the major educational themes and the categorization of implementation methods across included studies (Tables 2 and 3). These tables complement Table 1 and help clarify the linkage between the Results and Discussion.

**Table 2:** Aggregate key themes across included studies

**Key theme**	**No. of studies ** (n = 41)*	**Study No.**
Climate change & health (incl. climate-sensitive care)	18	3, 4, 7, 9, 15, 17, 19, 20, 21, 22, 23, 26, 27, 29, 32, 33, 36, 38
SDGs / sustainability / sustainable healthcare	14	3, 4, 6, 7, 8, 10, 12, 24, 34, 35, 36, 39, 40, 41
Pollution/waste & environmental toxins	4	8, 12, 35, 41
Food systems/ nutrition / planetary health diet	4	3, 6, 12, 24
Global health, health promotion & transformative learning	3	5, 18, 28
Planetary health concepts, systems thinking & interdisciplinarity	14	1, 2, 5, 11, 13, 14, 16, 18, 25, 26, 30, 33, 37, 38
Curriculum integration/reform & evaluation tools	19	9, 11, 15, 17, 18, 19, 20, 21, 22, 23, 25, 29, 30, 31, 32, 35, 36, 39, 40
Equity/justice & vulnerability (incl. Indigenous/transcultural perspectives)	4	5, 38, 39, 40

*One study may contribute to multiple themes. Key themes were coded for all included studies (n = 41) based on information explicitly reported in each study (e.g., objectives, program/intervention descriptions, and/or outcomes). Counts indicate the number of studies mapped to each umbrella theme; a study was counted if it addressed at least one of the listed elements, and multiple themes per study were allowed.

**Table 3:** Aggregate categorization of implementation methods across included studies

**Implementation method**	No. of studies (n = 41)*	**Study No.**
Online / e-learning	12	5, 6, 12, 16, 17, 18, 23, 28, 29, 30, 34, 37
Lecture/didactic	9	2, 6, 11, 17, 18, 20, 26, 30, 34
Group work/workshop	5	2, 9, 10, 20, 30
Practical/hands-on	5	2, 4, 7, 24, 41
Simulation / standardized patient / OSCE	4	25, 27, 30, 35
Serious game / game-based	2	37, 38
Flipped / peer teaching	2	24, 29
QI / project-based	2	34, 41
Blended learning	1	29

*One study may contribute to multiple methods.

Key themes were coded for all included studies (n = 41), allowing multiple themes per study (Table 2).

Based on Table 2, the most frequently reported content areas were climate change and health/climate-sensitive care and sustainability/SDG-related topics, together with cross-cutting themes such as systems thinking/interdisciplinarity and curriculum integration or evaluation tools. In contrast, biodiversity/ecosystem change, environmental toxins, and equity/justice-related content were reported less frequently.

Educational methods included diverse and combined lecture-style teaching [14, 23, 27, 28, 37, 45], group work [13, 16, 18, 37, 43], practical training [23, 29, 39], online learning [12, 24, 26, 29, 35, 45, 49], and simulations [19, 39]. Innovative pedagogies such as serious games [21, 44], flipped-classroom and peer-teaching approaches [32, 49], and blended learning [32, 33, 48] were frequently applied to enhance engagement and competence. Teichgraeber et al. [30] used the Objective Structured Clinical Examination method to assess students’ knowledge and practical skills.

Teaching materials and tools included online resources [12, 24, 26, 45], standardized patient simulations [39], and cooking workshops [29]. Recent studies introduced game-based learning [21, 44], reflective portfolios using Actor-Network Theory [13], and competency-based blended modules featuring structured self-reflection [32].

Interdisciplinary collaboration was prominent, with numerous studies involving experts from medical, environmental, and social science fields. Simon et al. [25] specifically researched the quality of PHE in German medical schools, emphasizing the importance of an interdisciplinary approach. Further work underscored collaborative reform movements and tool development, including student–faculty co-design of courses [33, 40], the Graduate Medical Education PHRC adaptation [35], and multi-institutional partnerships supporting trainee-led QI projects [47].

### Challenges and barriers

Institutional factors included difficulties in integrating content into existing curricula [36, 50, 51], time constraints [36], funding shortages [36], and resource limitations. Blanchard et al. [38] noted U.S. barriers such as curriculum space constraints, limited awareness, and knowledge gaps among educators. Similar constraints were identified globally: student advocates reported curriculum overload, insufficient faculty capacity, and lack of institutional accountability [40]; nursing leaders in Canada and Australasia confirmed faculty expertise gaps and absence of regulatory requirements [20, 46]. U.S. nursing faculty expressed low confidence in program preparedness and institutional support [41].

Cultural and social factors involved regional differences, variations in acceptability, and educator awareness. German final-year students displayed heterogeneous perceptions of their professional responsibility for climate-related health care and preferred interactive, practice-oriented teaching over mandatory examinations [34].

Structural factors included challenges in curriculum integration [50, 51] and unclear learning assessment indicators. Nordrum et al. (2022) reported that comprehensive curriculum revisions were necessary not only in Ireland but also globally. Recent European and North American studies emphasized that while elective courses proliferate [33], their limited mandatory integration and insufficient evaluation standards continue to hinder systemic adoption.

### Outcomes and impact evaluation

Learning outcomes included knowledge enhancement [13, 14, 28, 45], improved critical thinking skills [16, 25, 43], enhanced problem-solving abilities [16, 18, 25], and increased motivation for sustainable practices [22, 31]. Liu et al. [37] demonstrated a deepened student understanding of planetary health.

The new evidence further demonstrates measurable learning gains: for example, the pediatric CSHC module significantly increased planetary health literacy (p < 0.01) [32]; an Egyptian online course led to higher post-test scores (M = 16.69 vs. 12.41; p < 0.05) [49]; and the “Healthy Planet, Healthy People” board game produced substantial improvements in both attitudes (p < .001) and knowledge (p < .001) [21]. Serious-game interventions in the Netherlands also enhanced students’ recognition of professional responsibility (p = 0.003) [44]. Brazilian students participating in Actor-Network-based reflective exercises showed statistically significant increases in environmental interconnection and clinical relevance awareness [13].

Community and institutional impacts were evident, with McLean et al. [16] showing that student-produced products being used in policy proposals. Newer interventions extend these outcomes beyond the classroom: Canada’s TRASH-CAN initiative [47] has already generated 11 sustainability-focused QI projects, and German electives [33] reported transformative learning outcomes linked to student involvement. These findings were categorized as evidence beyond learner-level outcomes (institutional/system-level and community impacts) and are summarized in Table 4.

**Table 4:** Evidence beyond learner-level outcomes (program/institution/community level)

**Type of outcome beyond learner-level**	**Examples of evidence**	**Study No. examples**
Curriculum integration/reform efforts	Integration into multiple lectures; student-led curriculum reform; insufficient integration reported	18, 31
Curriculum mapping/audits/needs assessments	National/global surveys or audits of curricula; status of implementation across schools	11, 19, 30, 36, 40
Evaluation tools/metrics	Development/adaptation of PHRC metrics for program evaluation	39
Community engagement / QI initiatives	Trainee-led QI projects and implementation activities	41

Regarding evaluation methods for educational effectiveness, several studies reported short-term changes measured before and after interventions. For instance, a significant improvement in knowledge scores (p < 0.0001) was observed before and after lectures involving “Climate Wise” slides [45], self-assessment scores improved by 21–98% following seven flipped classroom sessions on the Planetary Health Diet [29], and emotional changes after planetary health lectures showed decreases in helplessness (−0.37) and disappointment (−0.35), along with an increase in confidence (+0.67) [31].

The newer interventions [13, 21, 32, 44, 49] provide additional quantitative validation of effectiveness using pre-post tests, Likert scales, and mixed-methods evaluation frameworks such as Kirkpatrick’s model [49] and the COM-B behavioral model [19].

However, longitudinal studies and randomized controlled trials evaluating the long-term effects of educational interventions were not found in the literature included in this review.

### Curriculum integration status

Integration methods included interdisciplinary subject integration [25, 27, 43], mandatory and elective course establishment [23, 28, 29], and targeted approach strategy development [17]. Simon et al. [25] specifically researched and discussed curriculum integration approaches in German medical education.

Recent studies [33, 34, 40, 42, 46] further elaborate on curriculum integration dynamics. Nationwide analyses in Germany [33] found that transformative learning outcomes were significantly higher in elective courses, while the GME PHRC initiative [42] expanded integration frameworks into postgraduate education. Qualitative interviews [34] highlighted medical students’ calls for communication-focused, practice-oriented learning rather than additional exams. In nursing contexts, integration often occurs through case-based content or Indigenous knowledge modules rather than standalone courses [46]. Finally, the TRASH-CAN program [47] represents a systems-level integration model, embedding planetary health competencies into institutional QI processes.

Successful practices, as illustrated by Zandavalli et al. [13], involved systematically introducing planetary health concepts with an emphasis on interconnectedness with nature and complexity.

Table 4 summarizes evidence beyond learner-level impacts, and Table 5 summarizes the framework-based grouping of outcomes.

**Table 5:** Outcome domains reported in included studies (framework-based grouping)

**Outcome domain**	No. of studies*	**Study No. examples**
Knowledge	18	1,2,3,4,6,8,12,16,17,18,23,24,27,29,33,34,37,38
Attitudes/Values (incl. emotions/satisfaction)	25	1,2,4,6,7,8,12,14,15,16,18,20,21,23,24,25,26,27,28,32,33,34,35,37,38
Skills/Competencies	5	5,6,18,25,29
Behavioral intention / Self-efficacy	16	1,3,4,5,6,12,16,17,18,23,26,28,29,32,35,38
Behavior/Practice	7	1,3,4,23,28,35,41
Institutional/System-level outcomes	18	7,9,10,11,13,15,18,19,20,21,22,23,30,31,36,39,40,41

*One study may contribute to multiple domains.

### Framework-based synthesis of outcomes

Reported outcomes were grouped using an evaluation framework (knowledge; attitudes/values; skills/competencies; behavioral intention/self-efficacy; behavior/practice; and institutional/system-level outcomes) (Table 5). Learner-level outcomes were most frequently reported, particularly changes in attitudes/values and knowledge. Evidence beyond learner-level outcomes was also identified (Table 4), including curriculum integration or reform efforts, curriculum audits/needs assessments, and the development of evaluation tools; however, these were often descriptive and less frequently evaluated using standardized indicators.

## Discussion

### Implementation status and current situation

PHE has been implemented in various regions, including Brazil, Australia, Germany, the United States, and Ireland. Notably, its integration into higher education institutions—particularly in medical and nursing schools—has been prominent. This trend likely reflects the growing necessity for healthcare professionals to understand the impacts of climate change and environmental issues on human health [55]. Publicly available MOOCs may play a crucial role in disseminating PHE. However, many educators are not well-versed in planetary health, which hinders the inclusion of this topic in curricula. This challenge appears to be common across educational settings globally [56].

In regions with high CCPI and PHDI rankings—such as Europe and North America—education on climate change and environmental issues is actively conducted, with planetary health being integrated into curricula. In contrast, in Southeast Asia and Africa, the introduction of PHE has been delayed due to limited educational resources, policies, and awareness among educators [11]. In these lower-ranked regions, raising awareness, providing educator training, and developing region-specific educational materials are crucial. The use of MOOCs may help reduce educational disparities across regions.

In this regard, the results of this review underscore that PHE is not merely a pedagogical challenge but also a reflection of broader structural inequities, including differences in national priorities, climate policy engagement, and institutional readiness. Addressing these disparities, therefore, requires multi-level strategies that include not only curriculum development but also policy support, investment in faculty training, and regionally adapted educational resources.

### Educational program components and implementation methods

The key themes of PHE programs include climate change and health, environmental hygiene, public health, and the Sustainable Development Goals (SDGs). These themes reflect the multifaceted and interdisciplinary nature of planetary health.

A combination of lectures, group work, hands-on training, online learning, and simulations has been implemented to accommodate diverse learning styles. In particular, innovative approaches such as virtual reality (VR) technology and culinary workshops have been introduced to enhance student engagement and promote practical learning.

VR environments have been reported to increase student motivation and engagement by making learning more interactive and enjoyable. This effect is particularly notable in vocational and engineering education, where VR helps simulate real-world scenarios [57].

Additionally, problem-based learning (PBL) is particularly effective in fostering higher-order thinking skills such as creativity and problem-solving. By incorporating PBL, students can engage more deeply with the content and develop the skills necessary for the 21st century [57].

Moving forward, integrating interactive, practical, and technology-enhanced educational approaches will be essential for cultivating professionals capable of addressing global health and environmental sustainability challenges.

In addition, the findings highlight that such approaches are most effective when they explicitly connect environmental changes to clinical decision-making and community-level health implications, suggesting that experiential and context-based learning may be a key condition for fostering planetary health competency development.

Beyond pedagogical approaches, the content mapping also provides insights into the contents emphasized within current PHE. As summarized in Table 4, the contents of PHE reported to date are frequently introduced through climate–health and sustainability/SDG-related entry points, while explicit coverage of other Earth-system changes (e.g., biodiversity/ecosystem change and environmental toxins) and equity/justice-oriented framing appears less consistently described. This pattern is noteworthy because planetary health competency development requires learners to understand multiple interacting environmental changes and their health pathways—not climate change alone—and to consider distributive impacts and vulnerability across populations. These findings also suggest a reporting issue: several programs describe “planetary health” broadly but provide limited detail on specific content domains, which hampers cross-study comparability. To strengthen curriculum development and evaluation, future programs and studies may benefit from articulating learning objectives and teaching materials using a transparent content framework—such as aligning course components with the PHEF core elements—and reporting content domains with sufficient granularity to enable synthesis and benchmarking across settings.

### Challenges and barriers

The main challenges in implementing PHE include difficulties integrating content into existing curricula, time and financial constraints, and insufficient educator awareness. These challenges are particularly evident in medical education, where curricula are already crowded. Cultural and social factors may serve as barriers. Prior research on global students’ awareness has shown substantial variability in recognition of greenhouse gases, influenced by scientific literacy and socioeconomic status, with notable differences across countries and regions [58]. To overcome these challenges, educator training programs and policy-level support are needed.

In addition, the current review indicates that the absence of clear competency frameworks and evaluation standards contributes to hesitation in adopting curricula. This suggests that internationally aligned planetary health competency guidelines may be essential for advancing systematic implementation.

### Outcomes and impact evaluation

As learning outcomes of PHE, improvements in knowledge, critical thinking, problem-solving skills, and motivation for sustainable practices have been reported. These outcomes suggest that PHE may contribute to students’ awareness and behavior change. However, current evaluations often focus on qualitative aspects, such as student feedback and knowledge acquisition [59]. Therefore, it is necessary to develop quantitative evaluation indicators to assess the effectiveness of PHE.

Additionally, very few studies have assessed whether behavioral changes or advocacy engagement are sustained over time, indicating a need for longitudinal follow-up and evaluation frameworks grounded in behavior change theory and implementation science.

### Curriculum integration status

The interdisciplinary subject integration approach reflects the inherently cross-disciplinary nature of planetary health. In particular, the case study from German medical education presents a concrete model for systematic curriculum integration. The strategic placement of mandatory and elective courses is an effective approach that ensures students acquire fundamental knowledge while enabling deeper learning based on individual interests. Furthermore, adopting targeted pedagogical strategies is crucial for achieving efficient learning outcomes within limited educational resources. Notably, successful cases of systematically introducing concepts that emphasize interconnectedness with nature and complexity are particularly significant. This approach appears highly effective in nurturing systems thinking and understanding of interconnectedness, which are essential characteristics of planetary health.

The findings suggest that successful curriculum integration requires the development of comprehensive educational frameworks, including faculty professional development and organizational support systems.

In particular, institutional leadership and accreditation bodies play a key role in determining whether planetary health becomes embedded as a core competency rather than an optional or advocacy-driven initiative. Strengthening institutional governance and aligning planetary health competencies with national qualification frameworks may therefore facilitate sustainable integration.

In line with the mission of “mainstreaming” public health within planetary health [60], PHE should move beyond elective or advocacy-driven initiatives and be embedded within core public health and professional curricula through institutional governance and system-level implementation. This direction is consistent with scalable and system-oriented examples identified in our review, such as MOOC-based delivery and trainee-led QI initiatives [12, 47].

### Implications for evaluating effectiveness beyond learner-level outcomes

While many studies reported short-term learner-level outcomes, evidence beyond the individual level (e.g., curriculum integration or reform, institutional policy change, and community engagement) was less consistently evaluated. Several studies described curriculum integration, institutional barriers/facilitators, or program-level tools (e.g., PHRC-related metrics), but formal evaluations of institutional policy change and sustained community-level impacts were limited. Future research should consider longer follow-up periods and evaluation approaches that capture program- and system-level changes alongside learner outcomes.

### Limitations

First, the review was limited to studies published in English and Japanese, which may have excluded relevant evidence from other language contexts. The geographical representation of the included studies was also focused on North America and Europe, potentially limiting the generalizability of the findings to other regions.

Second, the rapidly evolving nature of planetary health as an educational field means that this review, despite its broad scope, may not capture the most current developments, innovations, and challenges in this space. Ongoing updates and revisions to this scoping review will be necessary to maintain its relevance.

Third, because our search strategy was anchored to the term “planetary health” combined with “education,” relevant initiatives that align conceptually with planetary health but use alternative terminologies (e.g., EcoHealth/eco-health, One Health, or climate and health education) may have been underrepresented. This may have biased the observed geographical and disciplinary distribution of the included studies toward settings where the “planetary health” label has been more widely adopted. We used this narrower approach to maintain conceptual consistency with the PHE Framework, but future reviews could adopt broader search terms to capture a wider range of related initiatives.

Fourth, our search scope focused on higher education and health professional education, reflecting the aims of this review and where PHE has been most frequently reported to date. Accordingly, the disciplinary distribution should be interpreted within this scope, and initiatives in other disciplines may be underrepresented; future work could incorporate additional education and social science sources to capture wider disciplinary implementation.

In addition, because this review relied on published and accessible records, local or unpublished educational initiatives (e.g., internal curricula, institutional reports, or non-indexed regional programs) may not have been captured. This may have contributed to an overrepresentation of settings with stronger publication capacity.

## Conclusion

Current PHE predominantly focuses on medical education in high-income and upper-middle-income countries, with an emphasis primarily on short-term effectiveness evaluations. Future challenges include (1) expanding the scope to cover health and allied fields beyond medicine, as well as other disciplines; (2) conducting studies in countries with diverse socioeconomic backgrounds, especially low-income countries; and (3) implementing longitudinal studies and randomized controlled trials to assess long-term educational outcomes. These efforts are expected to contribute to a more comprehensive understanding of the effectiveness and impact of PHE, ultimately advancing solutions to global health challenges through education.

## Abbreviations

CDC: Centers for Disease Control and Prevention
PH: Planetary Health
PHE: Planetary Health Education
PHEF: Planetary Health Education Framework
PHDI: Planetary Pressures–adjusted Human Development Index
HDI: Human Development Index
UNDP: United Nations Development Programme
CCPI: Climate Change Performance Index
SDGs: Sustainable Development Goals
MOOC: Massive Open Online Course
VR: Virtual Reality
PBL: Problem-Based Learning
PCC: Patient–Concept–Context
PRISMA-ScR: Preferred Reporting Items for Systematic reviews and Meta-Analyses extension for Scoping Reviews

## Supplementary information

**Additional file 1:** **Supplementary Table S1.** Mapping of included studies to the Planetary Health Education Framework (PHEF) core elements (i–iv).

## Declarations

### Availability of data and materials

All data generated or analyzed during this study are included in this published article and its supplementary information files.
