# Supplementary material for: Current status and challenges of planetary health education: a scoping review
Source: Environ Health Prev Med. 2026 Apr 23;31:29. doi: 10.1265/ehpm.25-00437 (PMC13132659; doi:10.1265/ehpm.25-00437)
Supplement: Supplementary file 1 — Additional file 1: Supplementary Table S1. Mapping of included studies to the Planetary Health Education Framework (PHEF) core elements (i–iv). [file ehpm-31-029-s001.docx]

**Supplementary Table S1. Mapping of included studies to the Planetary Health Education Framework (PHEF) core elements (i–iv)**

PHEF core elements: (i) explicit linkage between environmental change and human health outcomes; (ii) systems thinking across ecological and societal determinants of health; (iii) solution-oriented approaches (e.g., mitigation/adaptation with health co-benefits, policy/governance, implementation/QI); (iv) equity/justice and transdisciplinary/interprofessional perspectives. Coding rule: “Yes” was assigned only when the element was explicitly described in the study’s objective, intervention, outcomes, or program description (as reported in Table 1); otherwise, “Not reported” was assigned.

| Study No. | Citation | PHEF-i | PHEF-ii | PHEF-iii | PHEF-iv |
| --- | --- | --- | --- | --- | --- |
| 1 | Zandavalli RB et al. (2024) | Yes | Yes | Not reported | Not reported |
| 2 | Christian M et al. (2023) | Not reported | Not reported | Not reported | Not reported |
| 3 | Weber A et al. (2023) | Yes | Not reported | Not reported | Not reported |
| 4 | Fülbert H et al. (2023) | Yes | Not reported | Yes | Not reported |
| 5 | Alonso Luaces M et al. (2021) | Not reported | Yes | Not reported | Not reported |
| 6 | Dumm M et al. (2023) | Not reported | Not reported | Not reported | Not reported |
| 7 | Huang A et al. (2024) | Yes | Not reported | Not reported | Not reported |
| 8 | McLean M et al. (2022) | Yes | Not reported | Yes | Not reported |
| 9 | Burch H et al. (2023) | Yes | Not reported | Not reported | Not reported |
| 10 | Duane B et al. (2024) | Yes | Not reported | Not reported | Not reported |
| 11 | Nordrum OL et al. (2022) | Not reported | Not reported | Yes | Not reported |
| 12 | Floss S et al. (2021) | Not reported | Yes | Yes | Not reported |
| 13 | Simon J et al. (2023) | Not reported | Yes | Not reported | Yes |
| 14 | Dambre et al. (2022) | Not reported | Not reported | Yes | Yes |
| 15 | Cygan H et al. (2024) | Yes | Not reported | Not reported | Not reported |
| 16 | Klünder et al. (2023) | Not reported | Not reported | Not reported | Yes |
| 17 | Luo OD et al. (2023) | Not reported | Yes | Yes | Not reported |
| 18 | Schmid J et al. (2023) | Yes | Yes | Yes | Not reported |
| 19 | Shea K et al. (2020) | Not reported | Not reported | Yes | Not reported |
| 20 | Liu I et al. (2022) | Yes | Not reported | Not reported | Not reported |
| 21 | Bates OB et al. (2022) | Yes | Not reported | Yes | Not reported |
| 22 | Blanchard OA et al. (2022) | Yes | Not reported | Yes | Not reported |
| 23 | Lemke D et al. (2022) | Yes | Not reported | Yes | Not reported |
| 24 | Rosenau N et al. (2023) | Not reported | Not reported | Not reported | Not reported |
| 25 | Teichgräber U et al. (2024) | Yes | Yes | Yes | Not reported |
| 26 | Schwienhorst-Stich EM et al. (2023) | Yes | Not reported | Not reported | Not reported |
| 27 | Ramkumar J et al. (2021) | Yes | Not reported | Not reported | Not reported |
| 28 | Hickman AC et al. (2022) | Not reported | Not reported | Yes | Not reported |
| 29 | Block S. et al. (2025) | Not reported | Not reported | Not reported | Not reported |
| 30 | Grieco F. et al. (2025) | Not reported | Not reported | Yes | Yes |
| 31 | Malani, K et al. (2025) | Not reported | Not reported | Yes | Not reported |
| 32 | Charlotte Flock et al. (2025) | Yes | Not reported | Not reported | Not reported |
| 33 | L. Nachira et al. (2025) | Yes | Not reported | Not reported | Not reported |
| 34 | Hanaa Saeed Elhoshy et al. (2025) | Yes | Yes | Yes | Yes |
| 35 | Wang et al. (2025) | Yes | Yes | Yes | Not reported |
| 36 | Tutticci, N et al. (2025) | Yes | Not reported | Yes | Not reported |
| 37 | Levett-Jones et al. (2025) | Yes | Yes | Not reported | Not reported |
| 38 | Stevens, M. et al. (2024) | Yes | Yes | Yes | Yes |
| 39 | Sarah Schear et al. (2024) | Yes | Yes | Yes | Yes |
| 40 | Astle, Barbara, et al. (2025) | Not reported | Not reported | Not reported | Yes |
| 41 | Best G.M. et al. (2024) | Yes | Not reported | Yes | Not reported |
